# Supplementary material for: Splice-Junction-Based Mapping of Alternative Isoforms in the Human Proteome
Source: Cell Rep. Author manuscript; Available in PMC 2020 Jan 15. (PMC6961840; doi:10.1016/j.celrep.2019.11.026)
Supplement: 2 [file NIHMS1546469-supplement-2.zip › DF1/PXD009737/TestisGluC/TestisGluC_22_SMC5_YQTVFQDLPNTLDE.pdf]

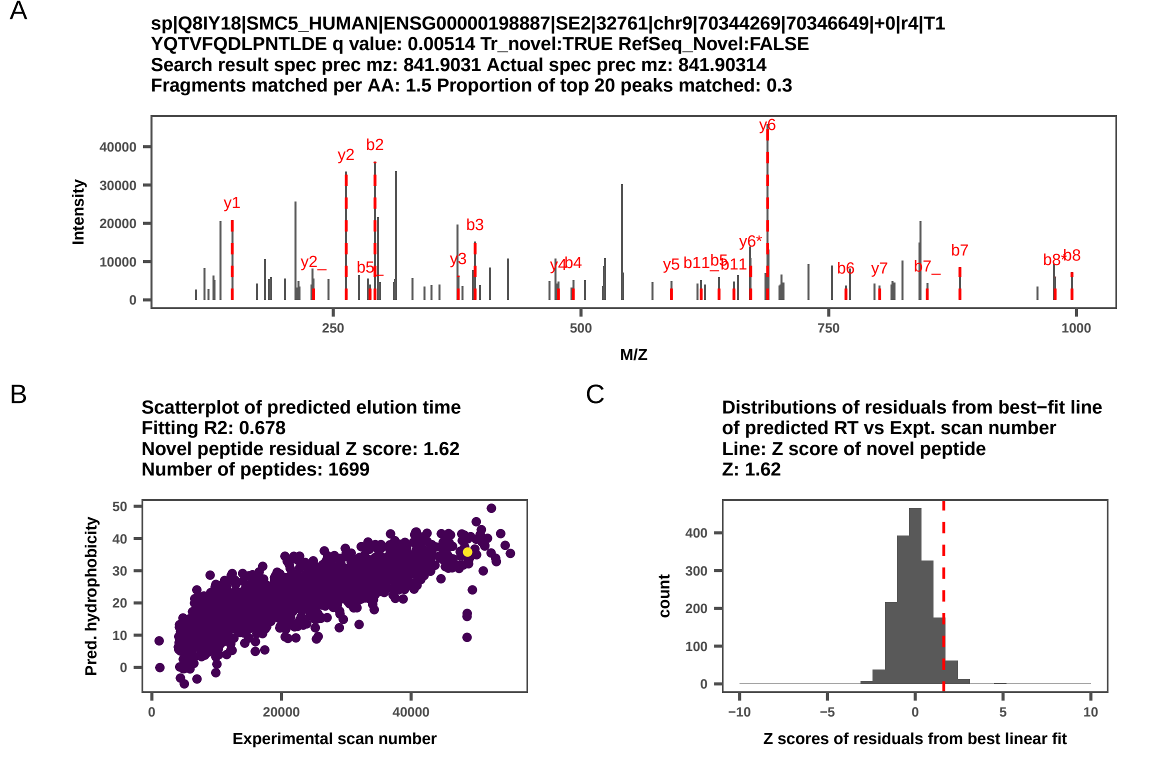

A
sp|Q8IY18|SMC5_HUMAN|ENSG00000198887|SE2|32761|chr9|70344269|70346649|+0|r4|T1
YQTVFQDLPNTLDE q value: 0.00514 Tr_novel:TRUE RefSeq_Novel:FALSE
Search result spec prec mz: 841.9031 Actual spec prec mz: 841.90314
Fragments matched per AA: 1.5 Proportion of top 20 peaks matched: 0.3
Intensity
40000
30000
20000
10000
0
y1
y2
b2
y2_
b5_
y3
b3
y4
b4
y5
b11
b5
_b11
y6*
y6
b6
y7
b7_
b7
b8*
b8
250
500
750
1000
M/Z
B
Scatterplot of predicted elution time
Fitting R2: 0.678
Novel peptide residual Z score: 1.62
Number of peptides: 1699
Pred. hydrophobicity
50
40
30
20
10
0
0
20000
40000
Experimental scan number
C
Distributions of residuals from best−fit line
of predicted RT vs Expt. scan number
Line: Z score of novel peptide
Z: 1.62
count
400
300
200
100
0
−10
−5
0
5
10
Z scores of residuals from best linear fit
